# High resolution annual average air pollution concentration maps for the Netherlands

**DOI:** 10.1038/sdata.2019.35

**Published:** 2019-03-12

**Authors:** Oliver Schmitz, Rob Beelen, Maciej Strak, Gerard Hoek, Ivan Soenario, Bert Brunekreef, Ilonca Vaartjes, Martin J. Dijst, Diederick E. Grobbee, Derek Karssenberg

**Affiliations:** 1 Department of Physical Geography, Faculty of Geosciences, Utrecht University, Utrecht, The Netherlands; 2 Global Geo Health Data Center (GGHDC), Utrecht University, Utrecht, The Netherlands; 3 National Institute for Public Health and the Environment (RIVM), Bilthoven, The Netherlands; 4 Institute for Risk Assessment Sciences (IRAS), Utrecht University, Utrecht, The Netherlands; 5 Julius Center for Health Sciences and Primary Care, University Medical Center Utrecht, Utrecht, The Netherlands; 6 Luxembourg Institute of Socio-Economic Research (LISER), Esch-sur-Alzette, Luxembourg; 7 Department of Human Geography and Spatial Planning, Faculty of Geosciences, Utrecht University, Utrecht, The Netherlands

**Keywords:** Atmospheric science, Risk factors, Environmental monitoring, Environmental chemistry

## Abstract

Long-term exposure to air pollution is considered a major public health concern and has been related to overall mortality and various diseases such as respiratory and cardiovascular disease. Due to the spatial variability of air pollution concentrations, assessment of individual exposure to air pollution requires spatial datasets at high resolution. Combining detailed air pollution maps with personal mobility and activity patterns allows for an improved exposure assessment. We present high-resolution datasets for the Netherlands providing average ambient air pollution concentration values for the year 2009 for NO_2_, NO_x_, PM_2.5_, PM_2.5absorbance_ and PM_10._ The raster datasets on 5×5 m grid cover the entire Netherlands and were calculated using the land use regression models originating from the European Study of Cohorts for Air Pollution Effects (ESCAPE) project. Additional datasets with nationwide and regional measurements were used to evaluate the generated concentration maps. The presented datasets allow for spatial aggregations on different scales, nationwide individual exposure assessment, and the integration of activity patterns in the exposure estimation of individuals.

## Background & Summary

Air pollution negatively affects health. Epidemiological studies have shown that long-term exposure to gaseous (e.g. NO_2_, NO_x_) or particle pollutants (e.g. PM_2.5_, PM_10_) may contribute to the development of asthma, lung cancer, diabetes, and cardiovascular diseases^1–6^. For an improved assessment of exposure to air pollution, capturing the spatial variation of street level air pollution concentrations is relevant. Land Use Regression (LUR) models based on measurements from monitoring stations and predictor variables such as traffic or land use have been shown to be suitable to explain the spatial variation in air pollution concentrations^7–10^. LUR models estimating individual exposure to air pollution have for instance been developed for the United States^11^, China^12^, metropolitan areas in Australia^13,14^ and within the European Study of Cohorts for Air Pollution Effects (ESCAPE)^10,15^.

Previous studies of health effects of long-term exposure used LUR models to calculate air pollution at selected locations, mostly front door locations of home addresses. This approach neglects human activity patterns which may be a fundamental omission as important air pollutants strongly vary in space and time. In case information is available for multiple locations such as home and work locations with associated residence times, or on activity patterns like commuting, estimating individual exposures to air pollution can be improved^16–18^. To enable this, concentration values at virtually any location and consequently air pollution data at a high spatial resolution and national coverage are required to calculate exposure for each individual within a cohort. In addition, such map data would enable calculating exposure for the entire population in a country.

We present a new consistent data set consisting of 5 metre resolution air pollution concentration maps covering the entire land surface of the Netherlands, an area of about 33 680 km^2^. We created these maps using the LUR models developed within the ESCAPE project^10,15^ for the Netherlands and Belgium, here only used for the Netherlands. The maps provide annual average concentration values for nitrogen (di)oxides (NO_2_, NO_2background_ and NO_x_, respectively), particulate matter with less than 10 *μ*m or 2.5 *μ*m (PM_10_ and PM_2.5_, respectively), and a proxy for elemental carbon (PM_2.5absorbance_). The concentration values calculated by the ESCAPE LUR models can be considered as long term average values as the models were identified using mean air pollution in 2009 and spatial contrasts are stable for multiple years. The LUR models were based on land use, traffic infrastructure, traffic intensity and population density. Predictor variables included aggregated values of these attributes calculated within circular buffers with radii between 25 m and 5 km.

To verify our mapping approach we compared the values of predictor variables calculated using our nationwide mapping techniques with those used to identify the ESCAPE LUR models^10,15^. To validate the maps, we used independent measurements of air pollution to assess the new concentration maps. A comparison with measurements obtained in the RUPIOH study^19^ for the municipality of Amsterdam resulted in an r^2^ of 0.22 for PM_2.5_, 0.38 for PM_2.5absorbance_ and 0.2 for PM_10_. A nationwide measurement dataset for NO_2_ from the TRACHEA study^20^ resulted in an r^2^ of 0.75. We also validated against air quality measurement data from the National Institute for Public Health and the Environment (https://www.rivm.nl/en/) obtained from stations across the Netherlands. The resulting r^2^ for NO_2_ is 0.85 and for PM_10_ is 0.57.

The spatially detailed air pollution concentration datasets enable health researchers to improve the assessment of the effects of spatial variability on human exposure and health. The data can be used to enrich cohorts with air pollution data either to assess the relation between air pollution and health or to take air pollution into account as a confounding factor. Feasibility of this method has been shown in studies investigating the relation between air pollution exposure and diabetes prevalence^6^ and lifestyle^21^. Concentration values can be obtained from the maps for cohorts of any size. Spatial aggregations over tracks followed by individuals, their close surrounding or over administrative areas can be straightforwardly computed with standard Geographic Information System (GIS) software.

## Methods

To generate high-resolution air pollution concentration maps we built upon the LUR models and datasets initially developed in the ESCAPE project^10,15^. First, we briefly describe the ESCAPE project and the therein developed LUR models covering the Netherlands. Then, the data preparation in the spatio-temporal modelling environment PCRaster^22^ and the application of the six air pollution concentration models over the Netherlands are described. The required software to calculate predictor variables and air pollution concentration maps is described in the ‘Code Availability’ section.

### The ESCAPE Land Use Regression models

After epidemiological studies established exposure response relationships in North America^23^ and European exposure estimates at that time were based on these results, the European Study of Cohorts for Air Pollution Effects (http://escapeproject.eu/) was initiated to investigate the contribution of long-term traffic-related air pollution to the state of health. The ESCAPE study covered 36 study areas in 15 European countries and was performed between 2008 and 2012. Standard operating procedures for measurements (http://www.escapeproject.eu/manuals/) and a standardised methodology for the assessment of long-term population exposure to air pollution were developed to investigate exposure-response relationships for e.g. respiratory and cardiovascular diseases.

For all European study areas, LUR models were developed based upon measured annual average concentrations. In the Netherlands, simultaneous measurements took place at 80 monitoring sites for NO_2_ and NO_x_, and 40 sites for PM. Regional background, urban background and traffic sites were selected. The Netherlands are located in the temperate climate zone of Western Europe (Cfb according to the Köppen-Geiger classification^24,25^) with a mean temperature of 10.1 °C and a mean amount of 851 mm precipitation. Three two-week measurement campaigns were performed in different seasons over the year 2009 (cold, warm and intermediate season) to capture seasonality. In addition, an ESCAPE background reference monitoring site was measuring pollutant concentrations the entire year. The data obtained from the three measurement campaigns were then averaged, adjusting for temporal trends using the continuous data from the reference monitoring site^15,26^. PM_10-2.5_ was not measured but calculated as difference between PM_10_ and PM_2.5_.

Geographic datasets of land use, traffic infrastructure and population density were available for all study sites and used to derive the predictor variables. Datasets presumably improving LUR models, such as street configuration or traffic speed^27^, were not available at European scale and therefore not considered. In the Netherlands, however, information on light-duty and heavy-duty traffic intensities were available and used in the model development.

For each monitoring site and geographic dataset, potential predictor variables were then computed using circular zones and various buffer sizes. Afterwards, for each pollutant the predictor variables explaining best the spatial variation in measured annual average air pollution concentrations were identified. The LUR models were then used to assess air pollution exposure for individual cohort participants, by calculating predictor variables and evaluating the LUR models at the front-door home address locations.

In the Netherlands, the ESCAPE LUR models explained 68% variability in the annual PM_10_ concentrations, 67% in the PM_2.5_ concentrations, 51% in the PM_10-2.5_ concentrations, 92% in the absorbance concentrations, and 86% in the NO_2_ concentrations. The lower model r^2^ for PM_2.5_ and PM_10_ compared to NO_2_ and PM_2.5absorbance_ is likely due to the smaller impact of local (traffic) sources on these pollutants. The variation related to large source areas and the transformation process in the atmosphere are more difficult to characterize with the empirical modelling approach. Certain sources such as agriculture or shipping were not evaluated in detail. The lower r^2^ for coarse PM is due to missing sources and lower precision of the measurements: coarse was calculated as the difference between PM_10_ and PM_2.5_.

The development of the LUR models and the evaluation of the model performance are explained in more detail for nitrogen (di)oxides^10^ and particular matter^15^. Table 1 shows the six LUR models for the Netherlands and Belgium study area that were also used to calculate the datasets presented here.

### Data sources for the ESCAPE project

The ESCAPE project used several geographic data sources to derive the predictor variables for the LUR models. Datasets in the project were available for all study areas in Europe, and supplemented with national data.

Traffic related predictor variables were calculated using the digital road network based on Eurostreets version 3.1, derived from the TeleAtlas MultiNet data set for the year 2008^15^. This dataset was used for the predictors holding the length (in m) of all roads and major roads in a buffer (RL and MRL, respectively), and the inverse distances (m^−1^) to the nearest road (IDC) or the nearest major road (IDM). The widths of roads were not explicitly given in the datasets but each centre of a road lane was specified as separate line segment.

For the Netherlands, local traffic network and traffic intensity data from the Netherlands Environmental Assessment Agency (http://www.pbl.nl/en/) were additionally used for predictor variables with traffic load (vehicles·day^−1^·m) on all roads (TL) and major roads (MTL), and heavy traffic load on all roads (HTL).

Land use information was derived from the CORINE land cover 2000 data set (https://land.copernicus.eu/pan-european/corine-land-cover). The CORINE land use categories were reclassified and used to create predictor variables holding areas (m^2^) for harbour (HAR), industry (IND) and areas with high and low density residential land (RES). Population density data (POP) was obtained from the INTARESE project dataset (http://www.integrated-assessment.eu/eu/).

Regional background estimates (BEO, BEX, BEP, BEA) were included for the study area as not all large-scale spatial trends in the air pollution concentrations could be explained by the potential predictor variables^10^. Concentration data obtained from 20 stations for nitrogen oxides and 10 stations for the other pollutants, respectively, were used to estimate four background concentrations by inverse distance weighted interpolation. A regional background estimate did not improve the PM_10_ model and was therefore not included.

### Spatially distributed modelling

The original ESCAPE project used vector data sources. The predictor variables and models were then calculated using vector-based Geographic Information System functions on a limited set of observations and home address locations. With this approach it was not feasible to calculate air pollution concentrations at all Dutch home address locations due to the extensive runtime of the vector-based operations. We therefore applied a raster-based modelling approach where first the vector input data sources were converted to 5 m resolution raster data, and predictor variables and LUR models were calculated from these data in a raster environment as well. This approach results in minor differences between the original variables calculated by a vector-based and variables calculated using the raster-based approach. The differences between both approaches are negligible as will be shown in the ‘Technical Validation’ section.

To calculate raster-based predictor variables and air pollution concentrations we used PCRaster^22^, an open-source environmental modelling platform providing a wide range of operations suitable to express spatio-temporal processes via the Python programming language (http://www.python.org/). To be usable in PCRaster, the geographic source datasets were translated using GDAL/OGR (http://www.gdal.org/). The scripts implementing the conversion steps from vector to raster data are included (Data Citation 1).

The datasets were created as follows: The population density dataset was rasterised with *gdal_rasterize*. For land use, first the CORINE dataset was rasterized with *gdal_rasterize*. Then, the CORINE land use classes were reclassified and recoded to individual raster maps holding industrial, harbour and residential areas. The total length of roads in each raster cell was calculated by intersecting a 5 m^2^ resolution fishnet grid with the road network to obtain all individual road segments per raster cell. Then, the lengths of each road segment in a raster cell were calculated in QGIS (http://qgis.osgeo.org). Finally, the length values were summed up per cell and the total road lengths were assigned as raster cell value.

### Calculation of the predictor variables

The rasterised data sources were used to calculate the predictor variables that are used in the LUR models. Different circular buffer sizes for land use and population density (with 1000 and 5000 metres) and for road lengths and traffic loads (with 25, 50, 500 and 1000 metres) were required to calculate the six LUR models. In total, 16 predictor variables with buffers were calculated. The summation of cell values covered by circular buffers of radii between 25 and 5000 metres was area-wide calculated in Python using the multiprocessing, PCRaster^22^ and NumPy^28^ modules. Depending on the size of the buffers the predictor maps were either calculated on a standard Linux workstation (40 cores Intel Xeon E5-2650, 128 Gb memory; buffers smaller than 1000 metres) or on the Dutch national supercomputer ‘Cartesius’ (buffers with 1000 and 5000 m radii).

For the inverse distances to road networks we calculated for each cell the distance of the cell centre coordinate to the nearest road using the *Distance* function from the GDAL/OGR module, and assigned the inverse distance as raster cell value.

The four regional background estimators were calculated by inverse distance weighting interpolation using air pollution measurements from 20 stations for nitrogen oxides and 10 stations for particulate matter. We first created raster maps with values at station locations and used these as arguments for the *inversedistance* function from PCRaster, with a radius of 100 km.

### Implementation and calculation of the LUR models

With each of the predictor variables available as individual raster file the LUR models can be implemented to compute the air pollution concentration maps. A PCRaster Python script illustrating the calculation of a LUR model is shown in Box 1. PCRaster provides a broad set of operations based on the map algebra and cartographic modelling concept^29^; these geospatial operations are available after importing the module of the same name. The required predictor variables are read from disk using the corresponding filenames by *readmap* operations (lines 4–6) and each assigned to new variables. These variables become thereby spatial data types that essentially hold information on geographic extent and discretisation, and two-dimensional arrays for the raster cell values. The LUR model for PM_10_ (see Table 1) itself is implemented in line 9. The arithmetic operators of the equation are executed for each raster cell, an approach comparable to other array programming languages. The resulting concentration values are then stored to a new geospatial dataset on disk (line 12). The five other LUR models are calculated in the same way.

**Figure 1:**
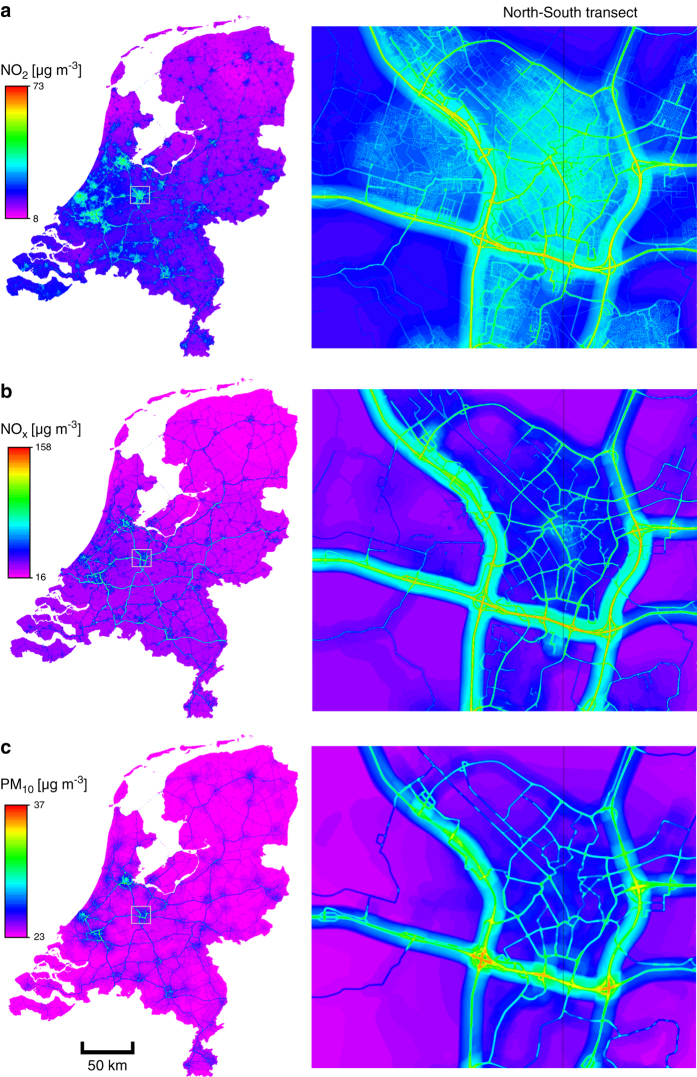
Air pollution concentration maps for the entire Netherlands with the box in the centre showing the municipality of Utrecht (left) and detailed maps for the municipality of Utrecht (right). The panels show concentrations for NO_2_ (**a**), NO_x_ (**b**) and PM_10_ (**c**).

**Figure 2:**
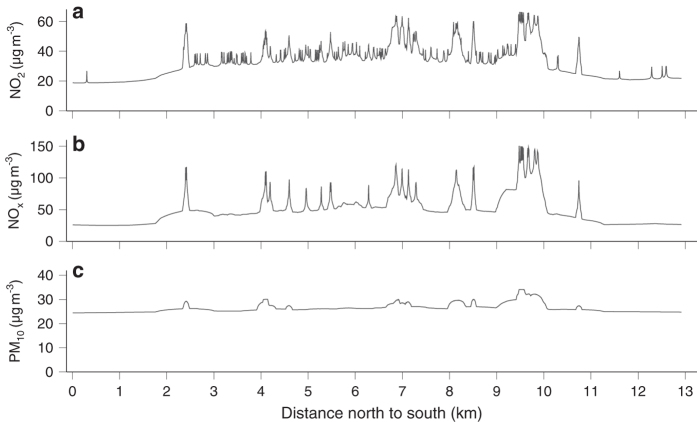
Air pollution concentrations along the North-South transect (shown in Fig. 1) in the municipality of Utrecht. The panels show NO_2_ (**a**), NO_x_ (**b**) and PM_10_ concentrations (**c**).

Examples for the resulting maps are shown in Fig. 1, illustrating the spatial pattern of air pollution concentration on national and municipality scale. Figure 2 shows the concentration values for a North-South transect through the municipality of Utrecht. The models include several small scale traffic predictors including inverse distance to roads that explain the gradient near roads. The increased concentration near intersections is due to higher 50 meter traffic buffers.

### Code availability

The PCRaster software package used to calculate the ESCAPE Land Use Regression models is open-source and can be executed on Linux, Windows and macOS. The PCRaster source code is available on GitHub (https://github.com/pcraster/pcraster/). Links to release packages for Windows, build instructions for Unices, reference documentation, online courses and information on research projects can be found at the project website (http://www.pcraster.eu). PCRaster version 4.2 is required to execute the models due to several recent code improvements for handling large datasets.

Additionally, Python (http://www.python.org/) version 2.7 (or 3.6) with the NumPy module^28^ version 1.7 (or higher) are required to calculate the land use regression models. The Geospatial Data Abstraction Library (GDAL, http://www.gdal.org/) version 2.2.4 (or higher) is required to execute the scripts that rasterise vector datasets, and to execute the scripts performing the distance to road calculations.

## Data Records

We provide the air pollution concentration maps resulting from the six LUR models, the Python scripts for data preprocessing, and the LUR model calculation. All content is included in a compressed file *nl_apc.7z* available through the public Zenodo repository (Data Citation 1).

Each concentration map is provided as individual file in the PCRaster binary file format. The PCRaster binary file format can be processed and visualised with the Aguila software^30^, which is included in the PCRaster package, common GIS applications such as ArcMap (http://www.esri.com/) or QGIS (http://qgis.osgeo.org), or converted to other raster formats or resampled to other grid cell sizes using the GDAL tools (http://www.gdal.org/) for further processing.

The maps cover the entire Netherlands with a 5× 5 m grid cell size (63500 rows, 54800 columns). The ESCAPE source datasets and the resulting raster datasets use the ‘Amersfoort/RD New’ (EPSG:28992) coordinate reference system. Concentration values for NO_2_, NO_2background_, NO_x_, PM_2.5_ and PM_10_ are given in *μ*g m^−3^, concentration values for PM_2.5absorbance_ are given in 10^−5^ m^−1^. The cell values represent average concentration values for the year 2009.

In addition to the spatial datasets we provide the Python scripts for the LUR model calculation and preprocessing of the predictor variables. The Python script *lur_models.py* performs the calculation of the land use regression models. The script *calculate_buffer.py* was used to aggregate cell values using circular buffers of various radii. The *calculate_distance.py* script performed the distance calculations to road networks. The *generate_landuse.py* script was used to generate predictor variables from the CORINE dataset. The regional background estimates were calculated with the *generate_regest.py* script, population densities were calculated with the *generate_population.py* script. Configuration settings valid for all scripts are specified in *settings.py*.

## Technical Validation

### Validation of the raster-based approach

We first calculated the predictor variables and LUR models on a small set of locations to evaluate whether the resulting concentration values obtained in a raster-based modelling approach are in agreement with the results obtained from the LUR models calculated using a vector-based approach. We used 8000 front door locations in the province of Utrecht, an area of about 66×59 km^2^. The locations were obtained from a cohort dataset previously used in the ESCAPE project.

Table 2 shows comparative statistics between the vector-based and raster-based approaches. Model results and measurements are compared in terms of the coefficient of determination (r^2^), the root mean square error

(1)RMSE=1N∑i=1N(oi−mi)2,

and the bias

(2)Bias=1N∑i=1N(oi−mi),

with *o*_*i*_ the observed and and *m*_*i*_ the modelled value for location *i*, and *N* the number of locations (*N*=8000).

Overall, there is a high agreement between values obtained from the newly calculated raster maps and the previously calculated concentrations, with most of the predictor variables resulting in r^2^ values above 0.9, and the air pollution concentration maps with r^2^ values above 0.98. Small deviations were expected due to the grid representation of the spatial domain, as each possible location within a raster cell obtains the value of the 5 m^2^ area.

The results of the comparison show that in general the chosen 5 m^2^ grid is appropriate to represent distance related attributes for the ESCAPE LUR models. In a following step, the raster-based approach was therefore applied to calculate concentration maps covering the entire Netherlands.

### Independent validation of the datasets

**Figure 3:**
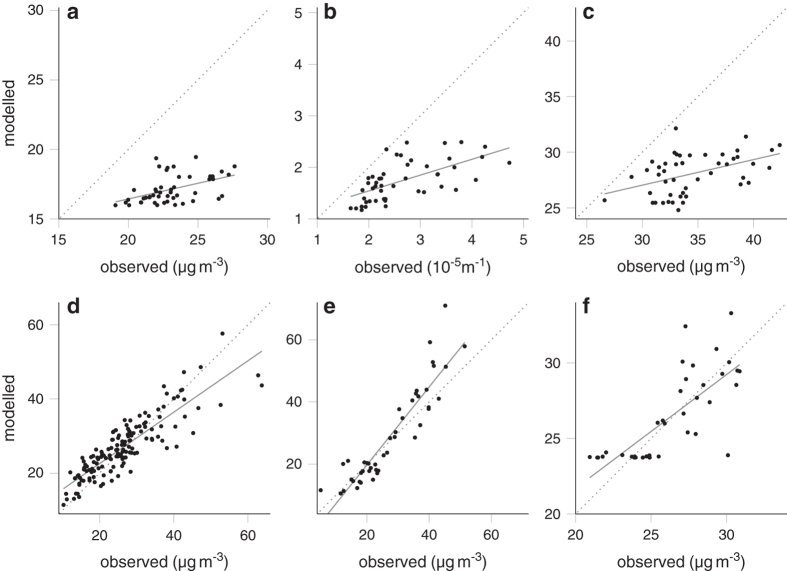
Agreement between modelled and measured concentrations. The panels show the scatterplots and linear model fits of PM_2.5_ (**a**), PM_2.5absorbance_ (**b**) and PM_10_ (**c**) for the RUPIOH dataset. (**d**) Shows NO_2_ for the TRACHEA dataset. (**e**) Shows NO_2_ and (**f**) PM_10_ for the LML dataset.

We used additional datasets with measured concentrations to assess the nation-wide datasets. The corresponding scatterplots are shown in Fig. 3 and the statistics are shown in Table 3.

The first dataset we used included monitoring data from the RUPIOH study^19^ (Relationship between ultrafine and fine particulate matter in indoor and outdoor air and respiratory health). Measurements of PM_2.5_, PM_10_ and PM_2.5absorbance_ were made at 48 locations spread over the city of Amsterdam between October 2002 and March 2004. Measurements were made directly outside the home, e. g. at balconies or in gardens, where feasible. The number of homes located at major roads and background sites was approximately equal. Annual average concentrations per site were calculated using data from a central reference site. The same measurement methods were applied as in the ESCAPE study used to develop the LUR models. We used this dataset because of the detailed spatial coverage of one city. NO_2_ was not measured in this study.

The LUR model predictions of the nation-wide dataset were moderately correlated with measured values and significantly underpredicted the measurements. Correlation was better for the component with more fine scale spatial variability (PM_2.5absorbance_). The low validation r^2^ for PM_2.5_ and PM_10_ in the Amsterdam validation dataset are likely due to the application of a national LUR model to a single city and to the modest spatial variation of PM within a city. Consistently, the validation r^2^ for PM_10_ was much higher (0.57 vs 0.22) in the national validation dataset (LML). The difference in validation raises concern with application of the model in a single city for PM_10_ and PM_2.5_. We further note that the validation dataset refers to 2002–2004 and the model was developed primarily based on measurements in 2009. There is also a downward air pollution trend in the Netherlands^31^, contributing to the lower estimates of the model compared to the measurements.

The second dataset involved measurements of NO_2_ made in the framework of the TRACHEA study (Traffic Related Air pollution and Children’s respiratory HEalth and Allergies^20^). Measurements were made simultaneously at 144 sites spread over the Netherlands using the same passive samplers as used in the ESCAPE study. Measurements were made during four 1-week periods spread over the seasons in 2007. Annual average concentration for 2007 was calculated.

Our NO_2_ LUR model predictions correlated well with the TRACHEA measurements. The large variability in concentrations makes it easier to compare model predictions with measured values. The agreement is affected by both small scale variation (traffic versus background) and regional variation.

We also used the nation-wide dataset provided from the Dutch Air Quality Monitoring Network (https://www.lml.rivm.nl/). Hourly measurements of NO_2_ and PM_10_ concentrations are recorded by the LML, which is maintained by the National Institute for Public Health and the Environment (https://www.rivm.nl/en/). There are four location types: urban, rural, traffic and industry. During the ESCAPE period from October, 1^st^ 2008 to April, 1^st^ 2011^15^, 49 stations provide measurement data. We excluded stations with more than 20% missing values in the data, resulting in 45 stations for NO_2_ and 38 stations for PM_10_. The NO_2_ and PM_10_ concentration are then averaged per measurement station over the ESCAPE period.

For the LML dataset we obtain an r^2^ of 0.85 for NO_2_, and an r^2^ of 0.57 for PM_10_. The lower correlation with measurements for PM_10_ compared to NO_2_ is due to the absence of a regional component in the ESCAPE model for PM_10_ (in contrast to PM_2.5_, NO_2_, PM_2.5absorbance_). In the international ESCAPE study, it was first attempted to explain measured variability by including small and large scale GIS variables and only then added regional variables to explain remaining variability. In the case of PM_10_, large scale population density entered the model, after which the regional background was no longer significant. For rural populations, the consequence may be that exposure contrast across the Netherlands is underestimated. Furthermore, the ESCAPE project did not include measurements specifically in intensive livestock rich areas and did not include GIS data representing farming emissions.

## Usage Notes

The six raster datasets presented can be opened, processed and visualised with the open-source PCRaster package. Any application making use of recent versions of the GDAL libraries can visualise or process the datasets for further statistical analysis, for example GIS applications like ArcMap (http://www.esri.com/) or QGIS (http://qgis.osgeo.org), or statistical software such as R (http://www.R-project.org). To reduce the data volume and allow for easier data handling, e.g. in local or regional studies, the datasets can be cropped or resampled by PCRaster, or converted to other raster formats by the GDAL tools.

The large file sizes of our datasets impose hardware requirements that may exceed present-day standard desktop computers. To allow for enriching cohort data with a subset of the air pollution maps, we also developed a web service facilitating access to various spatially aggregated derivatives of our raster maps. This functionality is demonstrated by the GGHDC exposure web portal (https://gghdc.geo.uu.nl/).

With our air pollution concentration map, several health research applications are feasible to investigate exposure-response relationships. The datasets can be used to extract concentration estimates for any coordinate in the Netherlands and arbitrary cohort sizes. In recent national health studies, for instance, associations between air pollution concentrations and smoking habits, alcohol consumption, physical activity and body mass index were investigated for 387 195 adults^21^ or to diabetes prevalence of 289 703 adults^6^.

Spatially fully distributed datasets also allow for a better representation and consequently incorporation of individual mobility patterns. By integrating road networks or GPS tracks and air pollution concentration maps, exposure along travel routes can be estimated. Alternatively, routes with minimal exposure can be calculated to suggest the healthiest routes for travel. Spatial aggregations using buffer sizes or administrative areas can be calculated to estimate exposure for persons with approximately known residence locations or movement patterns. As an additional dataset, the PM_coarse_ concentration can be calculated as difference between the PM_10_ and PM_2.5_ maps.

We are not able to provide the source datasets used to generate the predictor variables. However, the presented land use regression modelling approach is generic (e.g.^7,11,13,14^) and can be applied using other data sources as well. The Corine land cover data can be downloaded (https://land.copernicus.eu/pan-european/corine-land-cover). Predictor variables holding distance to nearest roads and road lengths could be calculated using OpenStreetmap datasets, e.g. provided by Geofabrik (http://download.geofabrik.de/). For traffic intensity based predictors, estimates based on a road type classification could be used. As the traffic intensity predictor variable received high weights in the Dutch LUR models, however, measurements might be preferable for this type of variables.

Our model included predictors starting from 25 m buffer sizes. We did not have very local predictor variables with the exception of inverse distance to roads. Our 5× 5 m estimates will therefore only gradually change with distance to traffic sources and should not be used to reflect very fine scale differences. The data can therefore not be used to interpret the absolute value of the concentration at a specific location, e.g. a specific home or school address. The models have been developed to characterise exposure contrast to be applied in research. The models include the main factors that explain spatial variation of air pollution, but individual locations may have deviating concentrations because of specific local factors not included in the model such as being in a narrow street canyon, stop-and-go traffic with higher emissions related to proximity to a traffic light or an exceptionally high fraction of old diesel cars. The concentrations should also not be compared strictly with air quality limit values. The monitoring instruments used to develop the ESCAPE LUR models were not formal reference instruments, though the difference with references instruments is limited. The models include main sources of air pollution with typically generic predictor variables. The models have not been developed to incorporate specific (industrial) point sources in a specific location. Monitoring in ESCAPE focused on residential addresses and as a consequence the models are less reliable in predicting on-road concentrations.

The reader should also notice that the values represent average values over the year 2009. The data set can be used for epidemiological studies that require estimates of personal exposures for other years or aggregated over multiple years (preferably in the range +/−10 years relative to 2009) as yearly mean values do not change considerably, but studies that use our dataset should take into account that there has been and continues to be a downward trend in concentrations while patterns in air pollution may change as a result of road construction or changes in traffic density on roads. The LUR models were developed with the intention to estimate long term exposure, the maps are therefore less suitable to estimate short term variation of air pollution concentrations. The ESCAPE project did not include measurements specifically in intensive livestock rich areas and did not include GIS data representing farming emissions. The model should therefore not be used to represent spatial variation related to farming emissions within local study areas. Concentration values in industrial areas might be underestimated as the Corine dataset does not distinguish between the types of industry. In addition, predictor variables with large buffer sizes are underestimated at the border zones as not all data in the neighbouring countries were available.

## Additional information

**How to cite this article**: Schmitz, O. *et al*. High resolution annual average air pollution concentration maps for the Netherlands. *Sci. Data*. 6:190035 https://doi.org/10.1038/sdata.2019.35 (2019).

**Publisher’s note**: Springer Nature remains neutral with regard to jurisdictional claims in published maps and institutional affiliations.

## Supplementary Material



## Figures and Tables

**Table 1:** Description of the Dutch LUR models.

Pollutant^a^	LUR model^b^
NO_2_	−7.8 + 1.18·BEO + 2.3e−05·POP_5000_ + 2.47e−06·TL_50_ + 1.06e−4·RL_1000_ + 9.84e−05·HTL_25_ + 12.19·IDC + 4.47e−07·HTL_25−500_
NO_2background_	3.21 + 0.74·BEO + 2.29e−05·POP_5000_ + 6.4e−07·IND_5000_ + 4.72e−07·HAR_5000_
NO_x_	3.25 + 0.74·BEX + 4.22e−06·TL_50_ + 6.36e−04·POP_1000_ + 2.39e−06·HTL_500_ + 71.65·IDM + 0.21·MRL_25_
PM_2.5_	9.46 + 0.42·BEP + 0.014·MRL_50_ + 2.28e−09·TML_1000_
PM_2.5absorbance_	0.07 + 2.95e−09·TL_500_ + 0.0029·MRL_50_ + 0.85·BEA + 7.90e−09·RES_5000_ + 1.72e−06·HTL_50_
PM_10_	23.71 + 2.16e−08·TML_500_ + 6.68e−06·POP_5000_ + 0.015·MRL_50_
Predictors with subscripted radii (m) are variables corresponding to the accumulated attribute value within the given circular buffer centred at the cell under consideration. Each model is calculated for each raster cell in the Netherlands.	
^a^Concentration of PM_2.5absorbance_ is given in 10^−5 ^m^−1^, the residual pollutant concentrations are given in *μ*g m^−3^. ^b^Regional background concentration estimates in *μ*g m^−3^ for NO_2_ (BEO), NO_x_ (BEX), and PM_2.5_ (BEP), and in 10^−5 ^m^−1^ for PM_2.5absorbance_ (BEA); traffic load (vehicles·day^−1^·m) on all roads (TL) and major roads (TML); heavy traffic load (vehicles·day^−1^·m) all roads (HTL); road length (m) of all roads (RL) and major roads (MRL); inverse distance (m^−1^) to all roads (IDC) and major roads (IDM); population density (inhabitants·m^−2^, POP); the surface area (m^2^) of industrial area (IND), harbour area (HAR), and residential area (RES).	

**Table 2:** Statistics of the modelled air pollution concentrations and predictor variables for 8000 house address locations in the province of Utrecht resulting from the comparison of the vector-based and raster-based modelling approaches.

Variable (units)	Buffer size (m)	r^2^	RMSE	Bias
NO_2_ (*μ*g m^−3^)	n/a	0.98	1.37	1.05
NO_x_ (*μ*g m^−3^)	n/a	0.98	1.9	1.13
PM_2.5_ (*μ*g m^−3^)	n/a	0.99	0.034	6.3e-3
PM_2.5absorbance_ (10^−5 ^m^−1^)	n/a	0.98	0.043	2.9e-2
PM_10_ (*μ*g m^−3^)	n/a	0.99	0.041	9.2e-3
TL (vehicles day^−1^ m)	500	0.95	1.24e7	8.43e6
RL (m)	1000	0.99	7.81e3	6773.61
HTL (vehicles day^−1^ m)	25	0.93	1731.53	396.77
	50	0.97	7111.19	2368.91
	500	0.98	5.94e5	−3.73e5
IND (m^2^)	5000	1.0	7.56e4	−7859.48
HAR (m^2^)	5000	1.0	3151.88	−42.71
POP (inhabitants m^−2^)	1000	1.0	14.25	−5.57
	5000	1.0	913.69	88.49
TML (vehicles day^−1^ m)	1000	0.99	3.89e6	1.59e6
RES (m^2^)	5000	0.99	2.42e5	−1.17e5
IDC (m^−1^)	n/a	0.85	0.026	4.3e-4
IDM (m^−1^)	n/a	0.91	4.1e-3	4.5e-5
BEO (*μ*g m^−3^)	n/a	1.0	0.06	−0.06
BEX (*μ*g m^−3^)	n/a	1.0	0.11	−0.1
BEP (*μ*g m^−3^)	n/a	1.0	1e-4	5e-5
BEA (10^−5 ^m^−1^)	n/a	1.0	4.64e-7	−7e-6
Units of RMSE and Bias are the same as the corresponding variable, their calculations according to Equations 1 and 2.				

**Table 3:** Statistics of the comparison of the independent measurement datasets and the modelled nation-wide raster datasets.

Dataset	Pollutant (units)	Number of sites	r^2^	RMSE	Bias
TRACHEA	NO_2_ (*μ*g m^−3^)	144	0.75	4.97	0.56^a^
RUPIOH	PM_2.5_ (*μ*g m^−3^)	48	0.22	6.24	−5.96^b^
	PM_2.5absorbance_ (10^−5^ m^−1^)	48	0.38	1.09	−0.91^b^
	PM_10_ (*μ*g m^−3^)	48	0.20	7.14	−6.42^b^
LML	NO_2_ (*μ*g m^−3^)	45	0.85	6.72	1.41^a^
	PM_10_ (*μ*g m^−3^)	38	0.57	1.99	0.14^a^
Units of RMSE and Bias are the same as the corresponding pollutant, their calculation according to Equations 1 and 2.					
^a^Not significantly different from 0 using t-test (P > 0.05).					
^b^Highly significant difference from 0 using t-test (P < 0.001).					

## Python script showing the fundamental steps to calculate the LUR models, here exemplary for PM_10_.

The preprocessed predictor variables are read from disk, the LUR model is calculated and the resulting air pollution concentration map is stored. Operations specific to PCRaster are marked in blue.
